# A retrospective analysis of the clinical characteristics of 207 hospitalized children with adrenal masses

**DOI:** 10.3389/fped.2023.1215095

**Published:** 2023-07-13

**Authors:** Kaiping Zhang, Ye Zhang, Yin Zhang, Min Chao

**Affiliations:** Department of Urology, Anhui Provincial Children’s Hospital/Children’s Hospital of Fudan University (Affiliated Anhui Branch), Hefei, China

**Keywords:** adrenal mass, adrenal hematoma, adrenal neuroblastoma, clinical characteristic, hospitalized children

## Abstract

**Objective:**

The detection rates of adrenal masses (AMs) have recently increased. The present study aimed to examine the clinical characteristics of these adrenal masses for guiding the clinical diagnosis and treatment among hospitalized children.

**Methods:**

The clinical data of AM cases admitted to our hospital from January 2014 to March 2023 were collected and analyzed retrospectively. The data included composition, sex, age, initial presentation, size and site of mass, functional tumor, intervention or surgery, pathological or clinical diagnosis, and imaging data.

**Results:**

A total of 207 hospitalized children were included. Among them, adrenal hematoma was the most common finding (53.6%), followed by adrenal neuroblastoma (36.2%). Most masses were larger-sized (51.2%) and non-functional (94.7%). We found that adrenal hematoma commonly occurred in a neonate or child with abdominal trauma. Most adrenal hematoma cases were found in male patients (63.1%), on the right side (71.2%), and with sizes <4 cm (73.9%). Adrenal neuroblastoma was commonly detected in male patients (56.0%), on the right side (66.7%), and with sizes ≥4 cm (85.3%). Moreover, the metastases were frequently explored at the time of diagnosis. In addition, there was no significant difference between ultrasound and computed tomography (CT) scans under suspicion of hematoma (*P* > 0.05). However, CT showed a priority over ultrasound in the diagnosis of neuroblastoma (*P* < 0.05).

**Conclusion:**

Most masses were non-functional and benign. Of these, adrenal hematoma was the most common type of pediatric AM, followed by adrenal neuroblastoma. They were both commonly found in male patients and on the right side. Neuroblastoma revealed a larger tumor size. Compared to cases of adrenal hematoma, cases of adrenal neuroblastoma required CT scans for further assessment.

## Background

As the frequent use of cross-sectional imaging is rising, adrenal mass (AM) is often detected incidentally in routine clinical practice. For adults, these AMs are asymptomatic masses (≥1 cm) that are discovered incidentally by imaging studies for reasons of non-adrenal disease. It has been reported that the incidence of AM is high in nearly 8% of autopsy series and 4% of CT scan series (1, 2). AM was commonly found in men older than 50 years, and bilateral AM occurs in 2%–10% of cases (3). In general, most discovered AMs are benign and poorly functional, which often do not show any physical signs of excess or insufficient hormone secretion. However, some adrenal carcinoma and functional tumors with varied clinical presentations should be properly diagnosed and treated due to potentially lethal lesions. Adrenal imaging contributes to differential diagnosis and tissue characterization.

An increased prevalence of AM is associated with age. For the elderly, the diagnosis and therapy of AM is still a more frequent task. However, the characteristic of AM in the pediatric population markedly differs from that in the adult population. In a young child, a soft-tissue mass should be highly suspect and indicate the possibility of adrenal neuroblastoma (AN) (4). Sometimes AN may be diagnosed on prenatal ultrasound (5). Adrenal hematoma (AH) is the most common AM in a neonate or child with abdominal trauma (6). Spontaneous resolution of variable AH will determine the management approach. In addition, adrenal adenoma is far less common during childhood than in adulthood. Most pediatric AMs are non-functional tumors. A palpable abdominal mass, abdominal pain, and/or hormonal hypersecretion symptoms are often the initial symptoms. AM in children can also be detected incidentally in recognizing extra-adrenal lesions. The purpose of this study is primarily to focus, with greater detail, on children with AM and explore the clinical features of pediatric AM, helping to assess AM and optimize the success of adrenalectomy.

## Methods

### Study design

A retrospective analysis of cases of children with AM from the past ten years, from January 2014 to March 2023, was conducted in our hospital. Only cases of AM diagnosed by imaging in our hospital were consecutively included in this study. Patients whose parents asked for discharge from our hospital for further treatment were excluded from this study, as were cases lacking patients' baseline characteristics and timely follow-up. The composition, sex, age, initial presentation, size and site of mass, pathological/clinical diagnosis, imaging data, and surgical intervention/observation were collected.

In the neonatal period, ultrasound is the primary imaging testing for the evaluation of AM, whereas CT or MRI is often required for differential diagnosis and further assessment, especially for critically ill patients. However, MRI was not routinely used owing to the need for sedation in these younger children, Determination of malignancy or benign tumor was based upon histological findings of surgical resections. The patients had regular follow-up times of 3–12 months after being discharged from the hospital. Follow-up of patients was conducted via telephone, subsequent out-patient visits, and other approaches. Besides, ultrasound has been widely used as a helpful tool for follow-up visits.

### Statistical analysis

In this study, data was performed by SPSS statistical software package (version 16) (IBM Corp., Armonk, NY, USA). The proportion of children was expressed as a frequency and percentage. The comparison of the two groups was performed with the chi-square test or Fisher's test. A *P*-value of less than 0.05 was considered statistically significant.

## Results

### Clinical characteristics

A total of 207 patients with AM were included in this study. Characteristics of AM are presented in Table 1. There were 96 cases confirmed by biopsy and 111 cases diagnosed by clinical features. After pathological/clinical examination, 111 cases were diagnosed with a hematoma, 55 with neuroblastoma, 20 with ganglioneuroblastoma, 6 with a cyst, 6 with ganglioneuroma, 3 with adrenocortical adenoma, 3 with pheochromocytoma, and one each with adrenocortical carcinoma, lymphangioma, and mature teratoma. Patients consisted of 122 boys (58.9%) and 85 girls (41.1%), aged between 1 day and 15 years. The size of AM ranged from 1 cm to 10 cm. Most cases were found with a mass larger than 4 cm (*n* = 106, 51.2%), non-functional masses (*n* = 196, 94.7%), and over 20 Hounsfield Units (HU) (*n* = 177, 85.5%). The detailed distribution is shown in Table 2.

**Table 1:** Characteristics of 207 adrenal masses.

Characteristic		*n* (%)
Sex	Boy	122 (58.9)
	Girl	85 (41.1)
Premature birth	Yes	4 (1.9)
	No	203 (98.1)
Birth weight	Low (<2,500 g)	11 (5.3)
	Normal (2,500–4,000 g)	173 (83.6)
	High (>4,000 g)	23 (11.1)
Mode of delivery	Cesarean delivery	68 (32.9)
	Vaginal delivery	139 (67.1)
Site of the tumor	Left	59 (28.5)
	Right	138 (66.7)
	Both	10 (4.8)
Tumor type	Benign	131 (63.3)
	Malignant	76 (36.7)
Size	≥4 cm	106 (51.2)
	<4 cm	101 (48.8)
Functional tumor	Yes	11 (5.3)
	No	196 (94.7)

**Table 2:** The detailed distribution of adrenal masses.

Clinical diagnosis	N	No. of resection/mass (%)				
		Incidentaloma	HU (≥ 20)	Large sized tumor (≥ 4 cm)	Functional tumor	Receiving operation
Adrenocortical adenoma	3	0 (0.0)	1 (33.3)	2 (66.7)	2 (66.7)	3 (100.0)
Adrenocortical carcinoma	1	0 (0.0)	1 (100.0)	1 (100.0)	1 (100.0)	1 (100.0)
Neuroblastoma	55	14 (25.5)	51 (92.7)	46 (83.6)	4 (7.3)	55 (100.0)
Ganglioneuroblastoma	20	7 (35.0)	18 (90.0)	18 (90.0)	0 (0.0)	20 (100.0)
Ganglioneuromas	6	3 (50.0)	3 (50.0)	5 (83.3)	0 (0.0)	6 (100.0)
Pheochromocytoma	3	0 (0.0)	3 (100.0)	3 (100.0)	3 (100.0)	3 (100.0)
Cyst	6	1 (16.7)	1 (16.7)	1 (16.7)	0 (0.0)	5 (83.3)
Hematoma	111	30 (27.0)	99 (89.2)	29 (26.1)	0 (0.0)	1 (0.9)
Lymphangiomas	1	0 (0.0)	0 (0.0)	0 (0.0)	1 (100.0)	1 (100.0)
Mature teratoma	1	1 (100.0)	0 (0.0)	1 (100.0)	0 (0.0)	1 (100.0)
Total	207	56 (27.1)	177 (85.5)	106 (51.2)	11 (5.3)	96 (46.4)

HU, Hounsfield units.

### Adrenal hematoma

A total of 111 patients were diagnosed with AH: 70 boys and 41 girls. It mainly occurred in the newborn period (55.9%). It was observed that 23 cases (20.7%), 79 cases (71.2%), and 9 cases (8.1%) had, respectively, an AH in the left adrenal gland, the right, and bilaterally, demonstrating a tendency of AH to be found on the right adrenal gland. There were 82 cases (73.9%) with a mass smaller than 4 cm. As for the risk factors of AH, 57 patients (51.4%) had experienced trauma prior to AH diagnosis, 42 cases (37.8%) possibly had a perinatal injury, and 10 cases (9.0%) had a history of infectious disease. Clinical presentations were always varied. There were 57 patients (51.4%) with blunt abdominal trauma, 23 patients (20.7%) with skin jaundice, and 7 patients (6.3%) with fever. Out of 111 patients, 110 patients received conservative treatment (Table 3). Follow-up examinations revealed a gradual regression of AH, even a complete absorption. Only one case underwent surgical treatment for the suspicion of adrenal carcinoma. A 27-day-old girl was admitted to the hospital with the chief complaint of jaundice disease. After 6 months of follow-up, a gradual regression was not detected in imaging. Moreover, a CT scan could not differentiate it from tumors. Postoperative pathology confirmed the lesion as a hematoma accompanied by calcification. Only one 4-year-old girl was diagnosed with both AN with masses larger than 4 cm. She was detected incidentally due to non-adrenal disease. After complete resection, the postoperative pathological result revealed a ganglioneuroblastoma at a localized stage.

**Table 3:** The clinical characteristics of 111 patients with adrenal hematoma.

Variable		*n* (%)
Age	≤1 m	62 (55.9)
	>1 m	49 (44.1)
Sex	Boy	70 (63.1)
	Girl	41 (36.9)
Site of the tumor	Left	23 (20.7)
	Right	79 (71.2)
	Both	9 (8.1)
Size	≥4 cm	29 (26.1)
	<4 cm	82 (73.9)
Risk factor	Trauma	57 (51.4)
	Perinatal injury	42 (37.8)
	Infectious disease	10 (9.0)
	unknown	2 (1.8)
Clinical presentation	Trauma	57 (51.4)
	Skin jaundice	23 (20.7)
	Fever	7 (6.3)
	Others	24 (21.6)
Intervention	Observation	110 (99.1)
	Surgical treatment	1 (0.9)

### Adrenal neuroblastoma

As shown in Table 4, there were 75 cases with AN confirmed by pathological diagnosis. The age of the patients ranged from 1 h to 9 years. A neuroblastoma in the right was explored in 50 cases (66.7%), left AN was found in 24 patients (32.0%), and only one patient had AN in both the right and left adrenal glands There were 64 cases (85.3%) with a mass larger than 4 cm. Among them, 84.0% of patients (*n* = 63) were ≤5 years old, and 56.0% (*n* = 42) were boys. On the pathological subtype, there were 55 cases of neuroblastoma (73.3%) and 20 cases of ganglioneuroblastoma (26.7%). Of tumor stage, most cases were diagnosed at a distant stage (49.3%, *n* = 37), 16% at a regional stage, and 34.7% at a localized stage. The most clinical presentation was abdominal pain (34 cases), followed by abdominal mass (12 cases), fever (11 cases), and others (8 cases). The remaining 21 cases (28.0%) were incidentally found for non-adrenal concerns, such as health examination, possible cholecystitis, abdominal hernias, and in consideration of other abdominal or urinary tract symptoms.

**Table 4:** The clinical characteristics of 75 patients with adrenal neuroblastoma.

Variable		*n* (%)
Age	≤1y	13 (17.3)
	>1y, ≤5y	50 (66.7)
	>5y	12 (16.0)
Sex	Boy	42 (56.0)
	Girl	33 (44.0)
Site of the tumor	Left	24 (32.0)
	Right	50 (66.7)
	Both	1 (1.3)
Size	≥4 cm	64 (85.3)
	<4 cm	11 (14.7)
Histology	NB	55 (73.3)
	GNB	20 (26.7)
Preoperative chemotherapy	Yes	27 (36.0)
	No	48 (64.0)
Tumor stage	Distant	37 (49.3)
	Regional	12 (16.0)
	Localized	26 (34.7)
Clinical presentation	Abdominal pain	17 (22.7)
	Abdominal mass	18 (24.0)
	Fever	11 (14.7)
	Incidentaloma	21 (28.0)
	Others	8 (10.6)

### Others

As for the other AMs, only one was adrenocortical carcinoma, whereas the remaining cases were benign. Most AMs occurred in boys and on the right adrenal gland. There were more cases with a mass larger than 4 cm. The initial presentations always varied, with abdominal pain/mass being the most common clinical presentation. These adrenal masses were discovered incidentally during imaging performed for clinical symptoms unrelated to adrenal disease. The detailed distributions are presented in Table 5.

**Table 5:** The clinical characteristics of 21 patients with the other adrenal masses.

Variable		Cyst (6)	GN (6)	Pheochromocytoma (3)	ACA (3)	ACC (1)	Mature teratoma (1)	Lymphangiomas (1)
Age	≤5y	3 (50.0)	3 (50.0)	0 (0.0)	2 (66.7)	0 (0.0)	0 (0.0)	0 (0.0)
	>5y	3 (50.0)	3 (50.0)	3 (100.0)	1 (33.3)	1 (100.0)	1 (100.0)	1 (100.0)
Sex	Boy	4 (66.7)	3 (50.0)	2 (66.7)	1 (33.3)	0 (0.0)	0 (0.0)	0 (0.0)
	Girl	2 (33.3)	3 (50.0)	1 (33.3)	2 (66.7)	1 (100.0)	1 (100.0)	1 (100.0)
Site of the tumor	Left	2 (33.3)	2 (33.3)	1 (33.3)	3 (100.0)	1 (100.0)	1 (100.0)	0 (0.0)
	Right	4 (66.7)	4 (66.7)	2 (66.7)	0 (0.0)	0 (0.0)	0 (0.0)	1 (100.0)
Size	≥4 cm	3 (50.0)	5 (83.3)	3 (100.0)	2 (66.7)	1 (100.0)	1 (100.0)	0 (0.0)
	<4 cm	3 (50.0)	1 (16.7)	0 (0.0)	1 (33.3)	0 (0.0)	0 (0.0)	1 (100.0)
Clinical presentation	Pain/mass	4 (66.6)	3 (50.0)	1 (33.3)	1 (33.3)	0 (0.0)	0 (0.0)	1 (100.0)
	Fever	0 (0.0)	1 (16.7)	2 (66.7)	0 (0.0)	0 (0.0)	0 (0.0)	0 (0.0)
	Incidentaloma	1 (16.7)	2 (33.3)	0 (0.0)	0 (0.0)	0 (0.0)	1 (100.0)	0 (0.0)
	Others	1 (16.7)	0 (0.0)	0 (0.0)	2 (66.7)	1 (100.0)	0 (0.0)	0 (0.0)

GN, Ganglioneuromas; ACA, Adrenocortical adenoma; ACC, Adrenocortical carcinoma.

### Diagnosis rate

Ultrasound is widely used for patients in the initial diagnosis and monitoring of AM. CT or MRI is often used for differential diagnosis and further assessment. Of the 111 patients diagnosed with AH, 22 of them only underwent ultrasound and 31 only underwent CT scan, whereas 58 patients received both ultrasound and CT scans. Of the 75 patients diagnosed with AN, 50 received both an ultrasound and a CT scan. We found that the initial diagnosis rates of ultrasound and CT scan in the diagnosis of AH were 86.3%% and 91.0%, respectively. There was no statistical difference (*P* > 0.05). However, CT scans showed a higher diagnosis rate of AN than ultrasounds (91.9% vs. 54.9%) and reached a significant statistical difference (*P* < 0.05) (Table 6).

**Table 6:** The initial diagnosis rates between ultrasound and CT.

	AH	AN	Others
Ultrasound	86.3% (69/80)	54.9% (28/51)	43.8% (7/16)
CT	91.0% (81/89)	91.9% (68/74)	64.4% (13/19)
χ^2^	0.957	23.185	
*P*-value	0.328	<0.001	0.182^a^

AH, Adrenal hematoma; AN, Adrenal neuroblastoma; CT, Computed tomography.

a Fisher's test.

## Discussion

AM has increasingly become a topic of interest in recent years. Most studies have focused on the characteristics of adult AM, and recommendations for diagnosis and treatment have been made based on these studies. However, the clinical characteristic of AM in a child differs greatly from that in an adult. The present study aimed to explore the clinical features that can assist in the diagnosis and therapy of pediatric AM.

AH is a relatively common disorder in children, frequently caused by blunt abdominal trauma, birth stress, anticoagulation therapy, bleeding disorders, infectious disease, and perinatal injury (7–9). Pediatric adrenal glands are larger than adult glands, and thus, children are more prone to suffer from abdominal trauma or birth stress. The clinical presentation related to adrenal insufficiency varies widely and it not only reflects the extent of the adrenal injury but the intensity of bleeding (10). The majority of AH diagnosis in children has been incidental as the course is asymptomatic (11). However, the clinical course of bilateral massive hemorrhage may be fatal if not timely diagnosed (12). With the aging of the hematoma, its gradually spontaneous shrinkage and even complete regression can be observed by imaging. Follow-up examination after a year detects adrenal calcification. To date, the diagnosis of AH is simple due to the increased availability of modern imaging techniques. The imaging findings mainly depend on the pediatric age and on the duration and intensity of bleeding (13). Ultrasonography is widely used for newborns and infants in the initial diagnosis and monitoring of AH. The primary choice for critically ill patients is CT scanning, and CT scans could also differentiate AH from malignant lesions (8). These patients often receive conservative treatment including anticoagulation therapy and adrenal infection prevention. In addition, the decision to pursue surgical treatment is made based on the clinical and laboratory markers of endocrinopathy and the radiological description of the adrenal lesion, which is highly suspected of massive hemorrhage and adrenal tumor.

Neuroblastoma (NB) is derived from the neural crest cells of the sympathetic nervous system (14) and is the most common extracranial solid tumor in children (15). NB accounts for 15% of all childhood cancer deaths, which could be explained by its aggressive nature and the presence of metastasis at the time of diagnosis (16). The adrenal gland is the common site of approximately 45% of NB cases (17). Approximately 40% of AN cases occurred in the first 3 months of life, and up to 90% of cases were confirmed by the age of 5 years (18). AN is more common in male patients than in female patients. The initial presentation is a palpable distention or abdominal pain attributed to the local effects of the primary tumor or its metastases. Diagnosis of AN is based on immunohistological results and imaging features. Histological analysis of the primary tumor should be performed as well as bone marrow biopsy for staging. Genetic analysis is now highly recommended for the influence of genetic abnormalities on prognosis (16). Ultrasound is the most common initial imaging used in the diagnosis of abdominal masses. CT or MRI scans are used for differential diagnosis and further assessment of AN. AN often reflects a cystic or solid mass adjacent to but separate from the kidney on ultrasound findings (19). Serial ultrasound imaging can help differentiate AH, which shrinks steadily over time. The typical findings of CT and MRI scans show a heterogeneous mass that crosses the midline and encases blood vessels but rarely invades them (20). MRI also shows superiority in evaluating spinal canal invasion. However, the need for sedation in younger children remains a disadvantage of MRI. The traditional open approach remains the mainstay of treatment for its aggressive nature and/or its diameter >4 cm. With the development of minimally invasive techniques, laparoscopic resection has become a good alternative to open surgery because of the smaller wound and faster recovery (21). Laparoscopic surgery is performed to safely resect AN with a diameter ≤4 cm and absence of vascular encasement (22). For larger or high-risk AN, a course of neoadjuvant chemotherapy may shrink the tumor and reduce vascularity before complete adrenal resection (23).

Adrenal cysts in children are mostly found incidentally. Symptoms frequently occur due to the effects of larger cysts or complications such as rupture and infection (24). Abdominal pain or palpable masses are often the typical presentations of large adrenal cysts. Owing to an internal liquid of adrenal cysts, ultrasounds reveal a round, well-defined anechoic lesion with thin walls, but cannot detect internal blood flow. On CT imaging, adrenal cysts appear round with a uniform density and no internal enhancement. Treatment depends on the underlying pathology, size, associated symptoms, and the occurrence of complications. Any functional, potentially malignant, or benign lesions of more than 5 cm in diameter deserve surgical treatment (25). Conservative management is a viable choice for small and benign cysts. Ganglioneuroma is a benign form of neural crest tumor in children. It is an incidental finding with no hormonal symptoms. Ultrasound examinations of ganglioneuroma show a well-defined, homogeneous, hypoechoic mass with some hyperechoic foci (26). On CT imaging, ganglioneuroma appears as a well-defined, encapsulated, hypoattenuating lesion. MRI is the best modality for evaluating intraspinal lesions. Treatment for ganglioneuroma is usually surgical resection, with an excellent prognosis.

Pheochromocytoma originates from chromaffin cells of the adrenal medulla and has an incidence of 0.2–0.5 cases per million children (27). It commonly secretes epinephrine in more than 95% of patients, leading to persistent or paroxysmal hypertension (28). Conversely, blood pressure may be normal despite high serum levels of catecholamines. CT or MRI scans are also the recommended mode of evaluation. The mainstay for treatment is surgical resection including conventional open and laparoscopic resection. Pediatric adrenocortical tumors (ACT) are rare but aggressive endocrine tumors, mainly comprising ACA and ACC. Carcinoma has an incidence of 0.2–0.3 per million patients aged under 20 years old, whereas the incidence of adenoma ranges from 0.3 to 0.38 per million children aged less than 15 years old (29–31). Compared to adult ACTs, most pediatric ACTs are functional, with symptoms of excessive androgen production (32). On imaging, adrenal adenoma appears as a well-defined and uniform mass and has homogeneous contrast enhancement. The imaging characteristic of carcinoma mainly presents a heterogeneous texture, resulting from intralesional hemorrhage, necrosis, and calcification in addition to local invasion and distant metastasis. Complete surgical resection remains the only potent treatment for pediatric ACTs.

In our study, AH was the most common AM followed by AN. We found that AH often occurs in a neonate or child with abdominal trauma. The clinical manifestation of AH varies widely. However, in many cases, the course of AH is asymptomatic. A follow-up examination is necessary to reassess the lesion. The patients in whom shrinkage of the tumor can be observed do not require surgery. AN is the most common adrenal solid tumor in children. Most cases are adrenal incidentaloma confirmed by the age of 5 years. For some cases, the initial presentation is a palpable distention or abdominal pain due to local effects of the primary tumor or the presence of metastases at the time of diagnosis. For larger ANs, the use of neoadjuvant chemotherapy can reduce tumor size and vascularity before complete resection. Though we collected medical records from the past decade, there were still some limitations. First, our study was retrospective. Second, only AM cases treated in our pediatric center were included, which could not represent the general condition. Finally, our study lacked a detailed and long-term follow-up. Therefore, a prospective, well-designed, and large-scale study should be performed.

## Conclusions

In our study, non-functional and benign AMs were commonly detected. We found that adrenal hematoma was the most common pediatric AM, followed by neuroblastoma. Most hematoma cases were found in male patients, on the right side, and were small-sized. A similar result was detected in neuroblastoma cases, except that in those cases, tumors were of a larger size. Compared to hematomas, adrenal tumors required CT scans for further assessment.

## Data Availability

The original contributions presented in the study are included in the article, further inquiries can be directed to the corresponding authors.
